# Final reflections

**DOI:** 10.1128/msphere.00345-25

**Published:** 2025-06-05

**Authors:** Michael J. Imperiale

**Affiliations:** 1 Department of Microbiology and Immunology, University of Michigan 1259, Ann Arbor, Michigan, USA

## EDITORIAL

“Messieurs, c'est les microbes qui auront le dernier mot.”

-Louis Pasteur

I still vividly remember the phone call I had 10 years ago with Tom Shenk and Barbara Goldman, in which they asked me to be the first editor in chief (EIC) of a new journal that ASM was going to launch: *mSphere*. I knew them both, Tom as a fellow DNA virologist for over 30 years and Barbara through my service as an editor of the *Journal of Virology* for the previous 9 years. It was, therefore, easy to have a frank conversation with two people I trusted very much. They were excellent salespeople and convinced me to sign on. I was especially enthusiastic about serving the ASM and its members. Our mission, in a nutshell, was to provide authors with a pan-microbial sciences, open access journal in which to publish their manuscripts, with the aim of accepting those whose importance fell within the top 50%.

My first task was to assemble an editorial team. Because of the broad scope of the journal, we decided that we would have a two-tiered editorial board, with senior editors (SEs), who would serve as the leadership team and be responsible for general areas of research, and editors, who would be appointed by the SEs to assist with those areas. Submitted manuscripts would be screened by the SEs, who would decide to handle the manuscript themselves or assign it to an editor. While my expertise was largely limited to virology, fortunately I had many friends and colleagues in other areas of the microbial sciences from whom I obtained suggestions in order to identify an outstanding group of SEs. Another thing that Barbara, Tom, and I decided was to target mainly mid-career scientists for this role. I began reaching out to people and tried to convince them to join me in this exciting endeavor. Tom’s and Barbara’s sales expertise must have rubbed off on me, because all but one of the people I initially contacted agreed.

We had a number of meetings early on during which we mapped out our philosophy for the journal and how we wanted it to work operationally. The ASM staff were amazing and just as excited as we were to get going. We began assembling the larger editorial team and preparing to receive our first submissions.

When we launched *mSphere*, our top priority was to provide authors with a positive experience. We structured our review process to minimize the number of hoops authors need to jump through, to provide maximum constructive feedback to authors, and to minimize the time to decision. These core values have driven us ever since then, and I think we have largely achieved these goals. It has been truly rewarding over the years to hear from authors who appreciated the way they were treated.

One of the factors that excited me about *mSphere* was Barbara’s and Tom’s encouragement to try experiments. The mSphere of Influence commentary series, along with the follow-up Full Circle minireviews from these same authors, has been awesome. It has been wonderful to see the different ways in which junior scientists have been motivated to pursue their careers.

Along the way, we also learned useful lessons from ideas that did not quite work out as planned. When we tried to democratize the AAM Fellow submission route in *mBio* by creating mSphereDirect, which allowed all authors to obtain their own reviews, we learned we had not fully appreciated how important it is to know more about reviewers. We therefore decided not to continue mSphereDirect, but I note that it paved the way for other ASM journals to implement similar peer review experiments.

In 2024, we initiated a collaboration with the American Geophysical Union to assemble a collection of papers relevant to One Health, Microbes, and Climate Change. This is exactly the type of cross-disciplinary initiative that fits perfectly into *mSphere’s* broad scope. We also began piloting a structured peer review process last year that we hope will help reviewers, editors, and authors as they contribute to the journal’s success. The pilot is ongoing.

In some ways, it is hard to believe that 10 years have passed. The world of traditional scientific publishing has been experiencing significant challenges: preprint servers, the continuing move to open access, predatory journals, and the advent of AI, to name a few. These have led to ever-increasing stresses on the financial model that has allowed ASM journals to thrive. Throughout it all, the SEs and I have strived to uphold the values that motivated and energized us from the start. *mSphere* has survived its childhood and adolescence (like dog years, journal years are not the same as human years) and is now entering into adulthood under the guidance of my friend Ira Blader. Ira has been with us as an SE since day one, and I think his vision for the future could not be more inspiring and timely, building on our successes and positioning the journal for the future. He also continues the tradition of having a New York Yankees fan as *mSphere* EIC.

I will end this editorial with some words of gratitude. I have learned so much from so many people who have helped me be a better EIC.

First, I wish to thank the authors who put their trust in a nascent journal, the reviewers who provided fair and critical feedback, and the editors who oversaw the reviews. You have all been participating in the extraordinary experiment that is *mSphere*, and I appreciate your support for the journal.

There are many people who accompanied me along the 10-year *mSphere* journey. The EICs of the other ASM journals have served as role models. Among them, I give special thanks to my friend Arturo Casadevall, whom I learned early on had lobbied to appoint me as the *mSphere* EIC and who has continued to provide wisdom and guidance over the years.

At ASM, our current staff, including Amanda Donaldson, Lorraine Clark, and Ani Mahapatra, have helped to shepherd us as the Society’s journals program has evolved to better serve its stakeholders. Nikki Glenn has been our peer review associate for many years and excels at holding the hands of authors, reviewers, and editors as they navigate the eJP system.

Our original team of Jasmine Wallace and Noel Lin were indispensable allies while the SEs and I learned how to edit a journal. Noel has become a great friend whose support I can always count on. I also want to give a special shout-out to Maisha Miles, who was the managing editor of *mBio* when we started and took a special interest in *mSphere*, joining us at all our meetings and providing helpful insights and advice. We were her adopted journal.

Amy Kullas was also always present to keep us aware of the ever-changing world of publication ethics; Adar Luckey and then Rachel Elliott have provided expert logistical support with meeting scheduling and travel; and Ellie Ghatineh, Stefanie Kowalski, Becky Zwadyk, and Miriam Day all worked tirelessly to keep the operation moving smoothly.

Finally, on the staff side, I have been fortunate to work with two incredible directors, Barbara Goldman and Melissa Junior. Their knowledge of the ins and outs of the publishing world is unmatched, and their enthusiasm for the *mSphere* experiment never waned. I have also been lucky to work with two totally supportive chairs of the Journals Committee, Tom Shenk and Pat Schloss, who also happen to have been terrific scientific colleagues. Speaking of support, one of our unwavering champions from the very beginning has been Stefano Bertuzzi, who took the time to meet regularly with us and share his feedback, ideas, and enthusiasm. Grazie mille, Stefano!

Last but not least, I need to thank the SEs. The original group consisted of Ira Blader, Melanie Blokesch, Patricia Bradford, Carolyn Coyne, Sarah D’Orazio, Paul Duprex, Trina McMahon, Aaron Mitchell, and Susannah Tringe. This team deserves special thanks for getting us off the ground. The way they all meshed, with a common vision for *mSphere*, was so critical, and I will always appreciate the time I spent working with this superb lineup of scientists. Over the years, there has been turnover, with Carolyn, Melanie, and Patricia leaving and Craig Ellermeier, Ana Fernández-Sesma, Paul Fey, Marcela Pasetti, and Vince Young joining us. Each and every one of the people named in this paragraph has contributed countless thoughts and immeasurable effort toward the success that *mSphere* now enjoys.

These 10 years have been eventful, exciting, and gratifying. As the great philosopher Yogi Berra said, “It’s tough to make predictions, especially about the future.” But I have no doubt that *mSphere* will continue to thrive and serve the microbial sciences community for many years to come.

